# Nematicidal Activity of 3-Acyltetramic Acid Analogues Against Pine Wood Nematode, *Bursaphelenchus xylophilus*

**DOI:** 10.3390/molecules22091568

**Published:** 2017-09-18

**Authors:** Hyo-Rim Lee, Sung-Chan Lee, Ji-Eun Lee, Seon-Mi Seo, Yong-Chul Jeong, Chan-Sik Jung, Mark G. Moloney, Il-Kwon Park

**Affiliations:** 1 Department of Forest Sciences, College of Agriculture and Life Sciences, Seoul National University, Seoul 08826, Korea; kazu21@naver.com (H.-R.L.); sungchan1225@empas.com (S.-C.L.); jie0815@snu.ac.kr (J.-E.L.); popcon24@naver.com (S.-M.S.); 2 Research Institute of Agriculture and Life Science, College of Agriculture and Life Sciences, Seoul National University, Seoul 08826, Korea; 3 Chemistry Research Laboratory, University of Oxford, Oxford OS1 3TA, UK; ycjchem@yahoo.co.kr (Y.-C.J.); 4 Division of Forest Insect Pests and Diseases, National Institute of Forest Science, Seoul 02455, Korea; csjung@korea.kr

**Keywords:** pine wilt disease, pine wood nematode, toxicity, lead compounds, 3-acyltetramic acid analogues

## Abstract

Among 98 3-acyltetramic acid analogues, compounds **1c**, **2c**, **2f** and **2g**, showed >90% nematicidal activity against the pine wood nematode *Bursaphelenchus xylophilus* at a 10 μg/mL concentration. The nematicidal activities of compounds **1d**, **1h**, and **2k** were a little lower at 88.0%, 85.8%, and 57.2% at a 10 μg/mL concentration, respectively. The nematicidal activity of emamection benzoate, widely used in Korea for the prevention of pine wilt disease, was 32.3% at a 10 μg/mL concentration. Other 3-acyltetramic acid analogues showed less than 30% nematicidal activity. A structure-activity relationship study indicated that the chain length of the C-acyl substituent was very important for high nematicidal activity. All active compounds had C_13_H_27_ or C_11_H_23_ acyl substituents, in two closely related groups with the common physicochemical properties of a polar surface area 57.6A^2^, PSA (polar surface area) 7.8–8.6% and ClogP (calculated partition coefficient) 5.1–5.9 and a polar surface area 75–84A^2^, PSA 11.1–11.6% and ClogP 4.7–5.1, respectively. Our study indicates that active 3-acyltetramic acid analogues could have potential as lead compounds for developing novel pine wood nematode control agents.

## 1. Introduction

Pine wilt disease is a serious problem in the pine forests of several Asian and European countries [1]. After pine wilt disease was first reported at Mt. Gumjung, Busan, this disease spread to several areas of the middle and southern parts of the Korean peninsula [2], leading to 1.74 million dead pine trees in 2015 [3]. Several kinds of methods have been developed for the control of pine wilt disease in Korea, and the Korean government has invested significant financial and human resources for several years to attempt to stop the spread of the disease [2]. Felling and fumigation of dead trees with metham sodium is used to kill the larvae of *Monochamus alternatus* or *Monochamus saltuarius* and the pine wood nematode. Aerial spraying with thiacloprid has been used to manage the adults of *M. alternatus* or *M. saltuarius*. However, the application of aerial spraying has decreased annually because of concerns over environmental contamination and toxicity to non-target organisms. Another control method is trunk injection of nematicides such as avamectin or emamectin benzoate, and this method is considered to be safer for the environment since the chemical agent is not widely dispersed. Avamectin and emamectin benzoate have been widely used for several years in Korea. Long and frequent usage of these nematicides increases the possibility of the emergence of resistance in the pine wood nematode, although there has been no research in this area as of yet.

Concern for the occurrence of resistant strains increases the need for the development of new and safer types of pine wood nematode control agents. Natural products derived from plants or microorganisms and their analogues provide good sources of lead compounds to develop new pest control agents. For this purpose, nematicidal activities of plant-derived natural products and their analogues against the pine wood nematode have been investigated in several studies [4,5,6,7,8,9]. 

In this study, we investigated the nematicidal activities of 98 3-acyltetramic acid analogues against the pine wood nematode, and developed their structure-activity relationships and physicochemical property–nematicidal activity relationships, in an attempt to find new alternatives for conventional nematicides.

## 2. Results and Discussion

### 2.1. Nematicidal Activities of 3-Acyltetramic Acids

**Figure 1:**
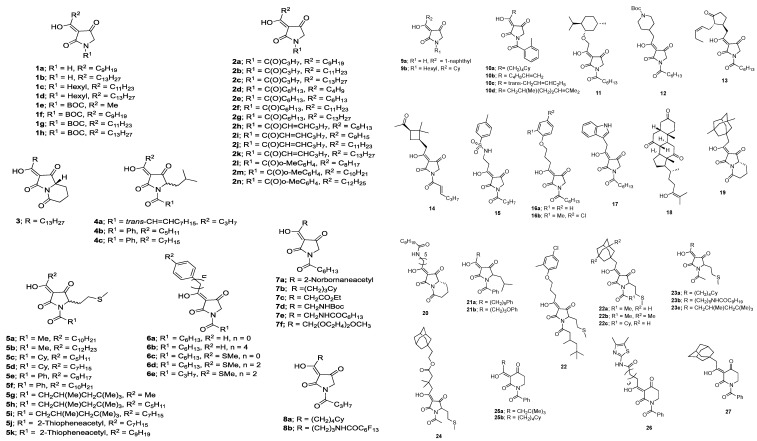
Chemical structures of monocyclic 3-acyltetramic acid analogues.

**Figure 2:**
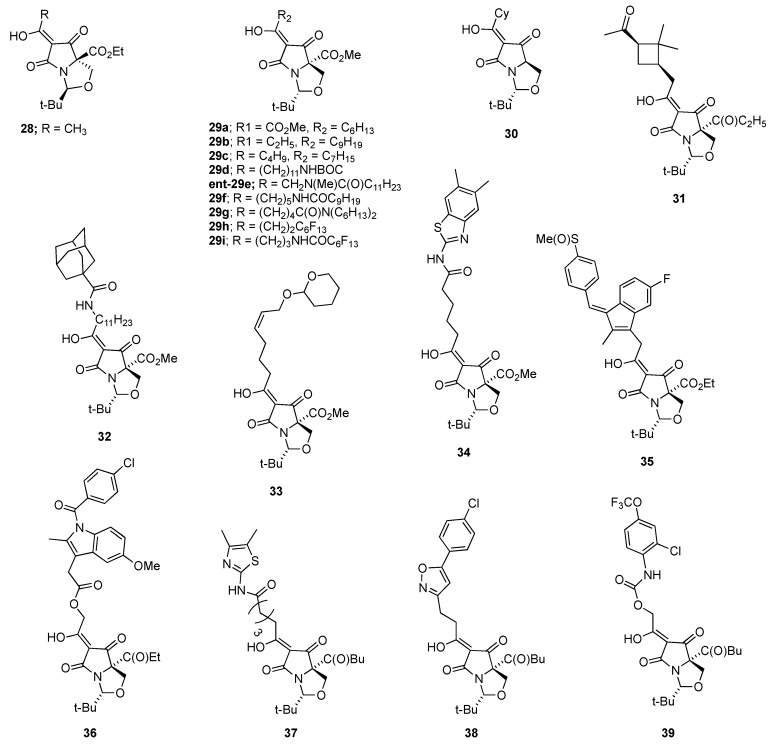
Chemical structures of 3-acyltetramic acid analogues.

The nematicidal activities of 3-acyltetramic acid analogues (Figure 1 and Figure 2) and emamectin benzoate are shown in Table 1. Among the test compounds, **1c**, **1d**, **1h**, **2c**, **2f**, **2g**, and **2k** showed very strong nematicidal activities against the pine wood nematode, and mortalities for compounds **1c**, **2c**, **2f** and **2g** were 99.6%, 100%, 95.8% and 98.9% at a 10 μg/mL concentration, respectively, while compounds **1d**, **1h**, and **2k** showed slightly lower nematicidal activities (85.8%, 88.0% and 57.2%) at a 10 μg/mL concentration, respectively. However, other 3-acyltetramic acid analogues showed less than 30% nematicidal activity. The nematicidal activity of 3-acyltetramic acids with ≥50% mortality at 10 μg/mL was tested at lower concentrations. Nematicidal activities of compounds **1c** and **2g** were 96.2% and 96.4% at a 5 μg/mL concentration, but their activities reduced to 15.0% and 6.3% at a 2.5 μg/mL concentration, respectively. Compounds **1d** and **2f** showed 80.2% and 76.4% nematicidal activities against the pine wood nematode at a 5 μg/mL concentration, but showed less than 40% mortality at a 2.5 μg/mL concentration, respectively. Other 3-acyltetaramic acids analogues showed <60% mortality at a 5 μg/mL concentration. This outcome is better than the nematicidal activities of emamectin benzoate, widely used for the prevention of pine wilt disease in Korea, which were 32.3%, 28.2% and 24.1% at 10, 5, and 2.5 μg/mL concentrations, respectively. Analogues with less than 30% mortality are not shown in Table 1. Recently, finding novel lead compounds inspired by bioactive natural compounds has been demonstrated to be an effective strategy for the development of pesticides [10]. Naturally occurring 3-acyltetramic acids are core structural skeletons with various biological activities such as magnesidin A [11], reutericyclin [12], integramycin [13], the melophlins [14] and the macrocidins [15]. Although antibacterial activities of 3-acyltetramic acids have been reported in a previous study [16], this is the first report of the nematicidal activities of 3-acyltetramic acid analogues against the pine wood nematode.

### 2.2. Structure-Activity Relationship

Understanding the structure-activity relationship is very important for developing lead compounds for novel pesticides. The chemical structures of compounds **1e**, **1f**, **1g**, and **1h** are very similar, but they differ in the identity of the acyl side chain, and among them, only compound **1h** showed strong nematidal activity against the pine wood nematode; compound **1h** has C_13_H_27_ at the C-acyl position while compounds **1e**, **1f** and **1g** have methyl, C_9_H_19_ and C_11_H_23_, respectively. Compounds **2a**, **2b**, and **2c** also showed similar structure-activity relationships, again with the only structural difference being the acyl side chain length. Compound **2c** with C_13_H_27_ at the R_4_ position showed strong nematicidal activity against the pine wood nematode, but compounds **2a** and **2b** with C_9_H_19_ and C_11_H_23_ on the side chain displayed very weak activity. All of compounds **2h**, **2i**, **2j** and **2k** have the same N-group, but different acyl side chain lengths, and the nematicidal activity of **2k** with C_13_H_27_ on the side chain was much higher than that of compounds **2h**, **2i** and **2j** with C_6_H_13_, C_9_H_19_, and C_11_H_23_ as R_4_ substituents, respectively. Compounds **1c** and **1d** have C_11_H_23_ and C_13_H_27_ on the side chain, and they all showed very strong nematicidal activities against the pine wood nematode. Another 3-acyltetramic acid group, compounds **2d**, **2e**, **2f** and **2g,** showed a similar result, and the nematicidal activities of compounds **2f** and **2g** with C_11_H_23_ and C_13_H_27_ on the side chain were much better than those of compounds **2d** and **2e** with C_4_H_9_ and C_6_H_13_. These results clearly indicate that an optimal chain length is essential for nematicidal activity against the pine wood nematode, and that biological activity is strongly correlated to chemical structure; this is developed further below. Seo et al. [17] investigated the structure-activity relationship of aliphatic compounds against the pine wood nematode, and also found that the chain length of the aliphatic compounds was very important for nematicidal activity; thus, among the alkanols and 2E-alkenols, the nematicidal activities of compounds with a C_9_–C_11_ chain length were much stronger than those of the other compounds with different chain lengths. In the 2E-alkenals and alkanoic acids groups, compounds with C_8_, C_9_ and C_9_, C_10_ chain lengths, respectively, exhibited strong nematicidal activity compared to compounds with C_12_–C_14_ chain lengths.

### 2.3. Physiocochemical Property–Nematicidal Activity Relationships

The 3-acyltetramate library comprises a series of substituted systems, varying in ring and nitrogen groups, as shown in Figure 1 and Figure 2. Of interest is that only a small subset of the examined compounds showed nematicidal activity against the pine wood nematode *B. xylophilus* at a 10 μg/mL concentration, and those compounds which were active (namely **2c**, **1c**, **2g**, **2f**, **1h**, **1d** and **2k**) were very similar in structure and possessed similar physicochemical properties (Table 2); thus, all active compounds had C_13_H_27_ or C_11_H_23_ acyl substituents in two closely related structural groups with the common physicochemical properties of a polar surface area (PSA) 57.6A ^2^, rel-PSA (relative polar surface area) 7.8–8.6% and ClogP 5.1–5.9 and a PSA 75–84A^2^, rel-PSA 11.1–11.6% and ClogP 4.7–5.1, respectively. This is a comparatively narrow range of parameters, at least in comparison with the full library, which has a much wider range of physicochemical properties (−0.43 < ClogD_7.4_ (calculated distribution coefficient at pH 7.4) < 5.3; −0.43 < ClogP < 6.1; 57 < PSA < 117A^2^; 7.8 < rel-PSA < 27.6% and 257 < MSA (molecular surface area) < 800A^3^). Of interest is that analysis of the physicochemical properties of acyltetramates in relation to their antibacterial activity has also been studied, and maximal activity also occurs for a narrow band of physicochemical descriptors, consistent with similar structures giving similar activities [16,18].

## 3. Materials and Methods

### 3.1. Chemicals

The chemical structures of 98 3-acyltetramic acid analogues are shown in Figure 1 and Figure 2. Their synthesis and characterization have been previously documented [16,19]. Emamectin benzoate (purity > 98%) was used as a positive control, and supplied form Syngenta Korea (Seoul, Korea).

### 3.2. Collection of the Pine Wood Nematode

Pine wood nematode *B. xylophilus* was supplied from National Institute of Forest Science, Seoul, Korea. We reared pine wood nematode on a lawn of *Botrytis cinerea* cultured on potato dextrose agar medium (PDA) in the dark at 28 °C. We extracted pine wood nematode by using Baermann funnel method [20] one day before bioassay.

### 3.3. Nematicidal Activity Test

To test the nematicidal activity of 3-acyltetramic acid analogues, test analogues were dissolved in ethanol (Daejung Chemical, Siheun, Gyeonggi-do, Korea) at a concentration of 1 mg/mL. 3-acyltetramic acid solutions (1 µL) were applied to the wells of a 96-well plate (JET Bio-Filtration co., Ltd, Guangzhou, China). Numbers of pine wood nematode in each well were about 50–150 nematodes (mixture of juvenile and adult nematodes, male:female:juvenile ≈ 1:1:2) in 99 µL of water. The total volume of the solution in each well was 100 µL, and the concentration of the test 3-acyltetramic acid analogues was 10 μg/mL. Ethanol (1 µL) and emamectin benzoate (Syngenta Korea, Seoul, Korea) were used as negative and positive control, respectively. In four adjacent wells (i.e., in a column) on the plate, pine wood nematodes were treated with 3-acyltetramic acid and a set of other 3-acyltetramic acid was placed in the wells of every next column. All experiments were replicated 4 times. We applied 3-acyltetramic acids randomly. The treated 96-well plates were stored in the dark at 25 ± 1 °C and 60% relative humidity. Mortality of pine wood nematode was determined after 48 h of treatment under microscope (65×). Nematodes were considered as dead if their bodies were motionless and straightened. 

### 3.4. Statistical Analysis

The percentages of mortality of pine wood nematode were transformed to arcsine square-root values prior to analysis of variance (ANOVA). Treatment mean values were compared and separated using Scheffe’s test. Statistical analyses were performed using IBM SPSS Statistics 23.0 (2015). Mean (±SE) values of untransformed data have been reported.

## 4. Conclusions

In this study, the nematicidal activities of 98 3-acyltetramic acids analogues against the pine wood nematode were evaluated. Among test compounds, compounds **1c**, **1d**, **1h**, **2c**, **2f**, **2g**, and **2k** showed very strong nematicidal activity. The structure-activity relationship study indicated that the chain length at the C-acyl position is essential for high nematicidal activity against the pine wood nematode. Further studies including the safety of active 3-acyltetramic acid analogues to human and non-target organisms, their formulations, and their modes of action are necessary to develop the practical use of 3-acyltetramic acid analogues as novel pine wood nematode control agents. 

## Figures and Tables

**Table 1:** Nematicidal activities of 3-acyltetramic acid analogues against *B. xylophilus.*

Compounds ^1^	Mortality (%, Mean ± SE, *n* = 4)		
	10 ^2^	5	2.5
**1c**	99.6 ± 0.7a ^3^	96.2 ± 0.9a	15.0 ± 3.0bc
**1d**	85.8 ± 3.5a	80.2 ± 6.8ab	35.5 ± 3.2a
**1h**	88.0 ± 7.3a	0.7 ± 1.3e	− ^4^
**2c**	100a	66.8 ± 8.1bc	6.3 ± 0.5cd
**2f**	95.8 ± 1.1a	76.4 ± 3.1b	10.8 ± 2.8c
**2g**	98.9 ± 1.9a	96.4 ± 1.8a	6.3 ± 3.6cd
**2k**	57.2 ± 18.8b	50.6 ± 10.1c	13.1 ± 3.9c
Emamectin benzoate	32.3 ± 2.7c	28.2 ± 3.9d	24.1 ± 4.3b
Control	0d	0e	0d
	F_8, 27_ = 80.208 *p* < 0.0001	F_8, 27_ = 156.542 *p* < 0.0001	F_7, 24_ = 41.067 *p* < 0.0001

Compounds ^1^ with >30% mortality at 10 μg/mL are shown. ^2^ μg/mL. ^3^ Means within a column followed by the same letters are not significantly different (Scheffe’s test). ^4^ Not tested.

**Table 2:** Physicochemical properties of active 3-acyltetramic acid analogues.

Compounds	Mw ^1^	MSA(A ^3^) ^2^	PSA(A ^2^) ^3^	rel-PSA (%) ^4^	ClogP ^5^	ClogD_7.4_^6^	H-D ^7^/H-A ^8^	RB ^9^
**1c**	366	674	57.6	8.55	5.12	4.51	1/3	15
**1d**	394	735	57.6	7.84	5.92	5.30	1/3	17
**1h**	410	721	83.9	11.6	5.13	4.26	1/4	14
**2c**	380	673	74.7	11.1	4.69	3.91	1/4	14
**2f**	366	674	57.6	8.55	5.12	4.51	1/3	15
**2g**	394	735	57.6	7.84	5.92	5.30	1/3	17
**2k**	378	642	74.7	11.6	4.77	3.92	1/4	13

Mw ^1^: Molecular weight. MSA ^2^: Molecular surface area. PSA ^3^: Polar surface area. rel-PSA ^4^: Relative polar surface area (%) = (PSA/MSA) × 100. ClogP ^5^: Calculated partition coefficient. ClogD_7.4_^6^: Calculated distribution coefficient at pH 7.4. H-D ^7^: Hydrogen bond donor count. H-A ^8^: Hydrogen bond acceptor count. RB ^9^: Rotatable bond count.
